# Feasibility of Optical Surface-Guidance for Position Verification and Monitoring of Stereotactic Body Radiotherapy in Deep-Inspiration Breath-Hold

**DOI:** 10.3389/fonc.2020.573279

**Published:** 2020-09-25

**Authors:** Patrick Naumann, Vania Batista, Benjamin Farnia, Jann Fischer, Jakob Liermann, Eric Tonndorf-Martini, Bernhard Rhein, Jürgen Debus

**Affiliations:** ^1^Department of Radiation Oncology, Heidelberg University Hospital, Heidelberg, Germany; ^2^Heidelberg Institute of Radiation Oncology (HIRO), Heidelberg, Germany; ^3^National Center for Tumor diseases (NCT), Heidelberg, Germany; ^4^Department of Radiation Oncology, University of Miami, Miami, FL, United States; ^5^Heidelberg Ion-Beam Therapy Center (HIT), Department of Radiation Oncology, Heidelberg University Hospital, Heidelberg, Germany; ^6^German Cancer Consortium (DKTK), Heidelberg, Germany; ^7^Clinical Cooperation Unit Radiation Oncology, German Cancer Research Center (DKFZ), Heidelberg, Germany

**Keywords:** stereotactic body radiation, Deep-inspiration breath-hold, surface-guided radiation therapy, Image-guided radiation therapy, precision radiation oncology, lung tumor, liver metastasis

## Abstract

**Background:**

Reductions in tumor movement allow for more precise and accurate radiotherapy with decreased dose delivery to adjacent normal tissue that is crucial in stereotactic body radiotherapy (SBRT). Deep inspiration breath-hold (DIBH) is an established approach to mitigate respiratory motion during radiotherapy. We assessed the feasibility of combining modern optical surface-guided radiotherapy (SGRT) and image-guided radiotherapy (IGRT) to ensure and monitor reproducibility of DIBH and to ensure accurate tumor localization for SBRT as an imaging-guided precision medicine.

**Methods:**

We defined a new workflow for delivering SBRT in DIBH for lung and liver tumors incorporating SGRT and IGRT with cone beam computed tomography (CBCT) twice per treatment fraction. Daily position corrections were analyzed and for every patient two points retrospectively characterized: an anatomically stable landmark (predominately Schmorl’s nodes or spinal enostosis) and a respiratory-dependent landmark (predominately surgical clips or branching vessel). The spatial distance of these points was compared for each CBCT and used as surrogate for intra- and interfractional variability. Differences between the lung and liver targets were assessed using the Welch *t*-test. Finally, the planning target volumes were compared to those of free-breathing plans, prepared as a precautionary measure in case of technical or patient-related problems with DIBH.

**Results:**

Ten patients were treated with SBRT according this workflow (7 liver, 3 lung). Planning target volumes could be reduced significantly from an average of 148 ml in free breathing to 110 ml utilizing DIBH (*p* < 0.001, paired *t*-test). After SGRT-based patient set-up, subsequent IGRT in DIBH yielded significantly higher mean corrections for liver targets compared to lung targets (9 mm vs. 5 mm, *p* = 0.017). Analysis of spatial distance between the fixed and moveable landmarks confirmed higher interfractional variability (interquartile range (IQR) 6.8 mm) than intrafractional variability (IQR 2.8 mm). In contrast, lung target variability was low, indicating a better correlation of patients’ surface to lung targets (intrafractional IQR 2.5 mm and interfractional IQR 1.7 mm).

**Conclusion:**

SBRT in DIBH utilizing SGRT and IGRT is feasible and results in significantly lower irradiated volumes. Nevertheless, IGRT is of paramount importance given that interfractional variability was high, particularly for liver tumors.

## Introduction

Focused delivery of high radiation doses to an extracranial tumor in few fractions is defined as stereotactic body radiotherapy (SBRT). It has become a commonly available and recognized treatment option for early stage primary tumors of or oligometastases from liver and lung primaries with a high rate of local control, often comparable to surgical resection (1–3). Due to technological advances in radiotherapy over the last decade, radiation plans with highly conformal dose distributions are widely available. This sculpted delivery of radiation dose is dependent on three-dimensional on-board imaging allowing image-guided radiotherapy (IGRT), which is now standard in modern linear accelerators. These improvements facilitate precise patient positioning and a safe and accurate characterization of dose deposition that are mandatory for SBRT. Nevertheless, moving targets are still challenging and respiratory motion management is the most crucial aspect for safe and effective utilization of SBRT (4).

In order to compensate for target motion, the International Commission on Radiation Units and Measurements (ICRU) introduced the concept of an internal margin to account for respiratory-induced changes in size, shape and position of a clinical target volume (CTV) (5). The addition of these internal margins to the CTV results in an internal target volume (ITV) to which further external margins for planning uncertainties are added to obtain a final planning target volume (PTV). This PTV is the volume that in the end receives the prescribed radiation dose. Yet, when this motion-encompassing approach is used for SBRT, the final PTV may become large or close to organs at risk (OAR), impeding the delivery of the high radiation doses needed for effective treatment. As a consequence, motion mitigation techniques were developed, which include: abdominal compression, beam-gating and breath-hold (6). Abdominal compression significantly reduces movement of the diaphragm and enables a good set-up accuracy for SBRT of liver and lower lung lobe targets in free-breathing (7, 8). For gated treatments an individual part of the respiratory cycle, usually the end-exhale phase, is chosen as a treatment window and further mitigates respiratory motion (4, 9). Tracking of targets with the beam is another method to manage respiratory motion, but requires real-time imaging during the treatment delivery (4, 10). The most reduction of respiratory movement, however, is achieved by breath-hold techniques that primarily target deep inspiration, which is tolerated longer than the end-exhale breath-hold phase and also carries less residual motion than gating of free breathing (4, 11).

For precise and accurate SBRT delivery, the reproducibility of deep inspiration breath-hold (DIBH) is crucial and has to be confirmed for several breath-hold cycles that are needed for a SBRT session. Depending on the PTV size, the beam-on time for SBRT typically varies between 2 and 5 min, despite already higher dose rates obtained by omission of the commonly used flattening filter (12). Reproducibility of DIBH can be assessed without additional radiation dose by either optical surface imaging solutions, such as AlignRT^TM^ (Vision RT, London, United Kingdom), or active breathing control devices, such as ABC^TM^ (Elekta, Stockholm, Sweden), which both reduce residual spatial uncertainties to 1–2 mm in standard radiotherapy of breast and lung cancer (13, 14).

Nevertheless, only limited data on SGRT and DIBH for SBRT is available. Here, we present our initial experience in utilizing a combination of SGRT and IGRT for patient positioning and treatment monitoring during SBRT of lung and liver targets.

## Materials and Methods

### Study Design and Patient Selection

Ten patients transferred for lung or liver SBRT to our department in 2018/19 were included in this pilot study. Only patients who could hold their breath for at least 30 s were eligible for study inclusion. All patients gave informed consent for an individualized SBRT approach using DIBH instead of treatment in free-breathing and abdominal compression which is standard of care. For monitoring of correct DIBH reproducibility the hard- and software tools of Vision RT Ltd. (London, United Kingdom) were used. These commercial solutions are officially approved and licensed for this purpose. The analysis was approved by the local ethics committee (S-063/2019).

### Radiotherapy Planning

Planning simulation was performed for all patients in vacuum cushion immobilization (BlueBAG BodyFIX^TM^, Innovative Technologie Völp (IT-V), Innsbruck, Austria). The edges of the cushion near to the target area were folded and smoothed to prevent shadowing and concealment of parts of the body surface for appropriate optical surface-guidance. Abdominal compression was not used to mitigate free breathing motion as it would prevent DIBH, recognition of the body surface by shadowing and could potentially modify the patient’s surface in an unreproducible manner. The AZ-733V Respiratory Gating System (Anzai Medical Co., Ltd., Japan) was utilized for registration of breathing motion and recording of time resolved, four-dimensional (4D) CTs. After 4D-CT acquisition, the respiratory belt was unstrapped and additional CT series in free-breathing as well as DIBH were recorded.

All CT data were transferred to our institutional treatment planning system (RayStation 6B, RaySearch Laboratories, Stockholm, Sweden). Target volumes were delineated using available contrast-enhanced imaging in both free-breathing and DIBH sequences. Planning target volumes (PTV) were generated in DIBH by adding a 3–5 mm safety margin to the clinical target volume (CTV). The CTV was created from the gross tumor volume (GTV) with an isotropic margin of 5–7 mm to account for microscopic spread. In contrast, the free-breathing PTV was created by a 2–3 mm expansion of an internal target volume (ITV) that integrated all motion information of the CTV extracted from the 4D-CT data. Treatment plans were calculated for both DIBH and free-breathing PTV, with the latter prepared as a precautionary measure in case of technical issues or patient-related problems with breath-hold.

### Treatment Delivery

**FIGURE 1:**
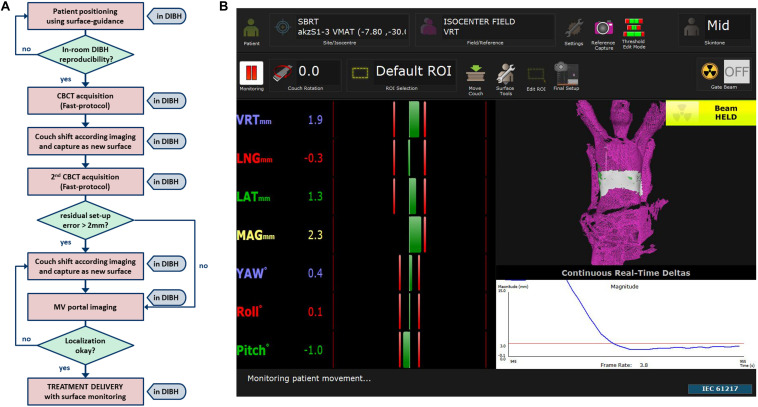
**(A)** Work flow of patient set-up and localization until treatment delivery together with **(B)** an example screenshot of one of our SBRT patients showing a typical region of interest (ROI) placed to the lower thorax for DIBH monitoring of a liver target.

The SBRT workflow of this study incorporates positioning and monitoring of patients in DIBH with surface-guidance (Figure 1A). A screenshot of the software (AlignRT^TM^ version 5.1.1, Vision RT, London, United Kingdom) used for DIBH positioning and monitoring is shown in Figure 1B.

In brief, the software allows for accurate patient set-up in DIBH and a radiation-free real-time feedback of DIBH positioning during the treatment session. Reproducibility of DIBH position was validated in-room by one to two repetitions. To verify the surface-guided position, a fast cone-beam computed tomography (CBCT), which lasts a breath-hold of 30 s, was acquired in DIBH. After registration to the planning CT, the couch was moved in DIBH accordingly and this new image guided position directly captured as a new surface reference in the SGRT software. To validate the reproducibility of tumor position in different breath-hold sequences, an additional fast CBCT was acquired. If a new registration resulted in shifts >2 mm, the couch was moved in DIBH as needed and the reference surface updated. Finally, orthogonal 2D-MV portal imaging was acquired in DIBH to confirm correct isocenter positioning.

After correct patient positioning the treatment was delivered in DIBH by a linear accelerator (Versa HD, Elekta, Stockholm, Sweden) using a flattening filter free (FFF) technique to reduce beam-on time whenever possible. We decided to set the individual defined region of interest (ROI), for which the SGRT software calculates differences in patient’s actual surface and the reference surface, to the lower thorax to measure thoracic motion during DIBH and to avoid large distances between the treatment isocenter and the ROIs’ centroid in liver SBRT. During treatment delivery the maximum allowed position error was 3 mm and 2° for translational and rotational differences, respectively. For patients’ comfort the treatment delivery was also stopped every 20–40 s to allow for breaks and prevent patients from becoming out of breath. The overall treatment time including positioning and IGRT varied between 20 and 60 min depending on various patient specific factors concerning breath-hold (maximum tolerated breath-hold duration, time until normalization of the respiratory rate, reproducibility of breath-hold) resulting in different numbers of breath-holds needed.

### Data Analysis

Differences in couch coordinates were assessed during the course of daily CBCT imaging (intrafractional) and compared to the derived shifts from subsequent treatment days (interfractional). For each patient and CBCT, a static, breathing-independent anatomic landmark (mostly Schmorl’s nodes or enostosis of the spine at the level of the gross tumor) and a moving, breathing-dependent landmark (such as clips near the liver target lesion or a pulmonary vessel branching adjacent to the lung target lesion) were defined and their intra- and interfractional positions compared.

### Statistical Analysis

Prism 7.04 software (GraphPad, San Diego, CA, United States) was used to perform statistical analysis and graphical plotting of results. Results were considered significant when the two-tailed *p*-value was less than 0.05. D’Agostino and Pearson omnibus K2 normality tests suggested a Gaussian distribution for the continuous variables of the vector length of position correction and spatial distance of fix and moving points, gross tumor volumes (GTV) and planning target volumes as well as the differences in planning target volumes in free-breathing and in DIBH for the same patient. Statistical differences between volumes of lung and liver targets were assessed using unpaired *t*-tests with Welch correction (Welch *t*-tests), which are more reliable for populations with unequal sample sizes and different variances than Student’s *t*-test. Paired *t*-tests were conducted for comparison of PTVs in DIBH and in free-breathing of the same patients. Frequencies of needs for a second position correction were grouped by target location and analyzed in a 2 × 2 contingency table and differences analyzed using Fisher’s exact test.

## Results

As a proof of concept analysis, we performed SBRT in DIBH in a total of 10 patients and for 41 treatment fractions using a combination of CBCT image- and surface-guidance for position verification and monitoring of DIBH. Details of our workflow are depicted in Figure 1 and explained in the methods section. Targets were either a primary tumor or single metastasis of the lungs and liver in 3 and 7 patients, respectively. Details on patients’ demographics and treatment delivery are shown in Table 1. Lung targets were mostly small peripheral lesions and could be treated in 3 fractions. In contrast, the liver tumors differed in size and some lesions were adjacent to organs at risk such as the gastrointestinal tract requiring individual dose fractionations between 3 and 8 fractions.

**TABLE 1:** Patient demographics and treatment patterns.

**No.**	**Sex, age**	**Primary tumor**	**Target lesion**	**Irradiation technique**	**Dose and fractionation**	**Prescription isodose†**
L1	f, 38	Breast cancer	Lung metastasis	3DCRT	15 Gy ×3	65%
			(segment 8 left)	(8 beams, FFF)		
L2	f, 69	NSCLC	primary	3DCRT	15 Gy ×3	65%
			(segment 3 left)	(8 beams, FFF)		
L3	f, 59	NSCLC	Lung metastasis	3DCRT	15 Gy ×3	65%
			(segment 9 right)	(9 beams, FFF)		
H1	f, 56	Breast cancer	Liver metastasis (segment IVa/VIII)	IMRT – VMAT	7.5 Gy ×8	80%
				(3 arcs, FF)		
H2	m, 76	HCC	Liver metastasis	IMRT – VMAT	15 Gy ×3	65%
			(segment VII)	(2 arcs, FF)		
H3	m, 74	HCC	Liver metastasis (segment VI)	IMRT – VMAT	7.5 Gy ×8	80%
				(2 arcs, FFF)		
H4	m, 48	Pancreas	Liver metastasis (segment VI/VII)	IMRT – VMAT	15 Gy ×3	65%
				(2 arcs, FFF)		
H5	m, 53	Prostate	Liver metastasis (segment VI)	IMRT – VMAT	6 Gy ×6	80%
				(2 arcs, FF)		
H6	m, 62	Pancreatic NEC	Liver metastasis (segment VIII)	IMRT – VMAT	9 Gy ×3	80%
				(2 arcs, FF)		
H7	f, 54	Breast cancer	Liver metastasis (segment IVa)	IMRT – VMAT	15 Gy ×3	65%
				(2 arcs, FFF)		

*^†^PTV-encompassing isodose. 3DCRT, 3D conformal radiotherapy; FF, flattening filter; FFF, flattening filter free; HCC, Hepatic cellular carcinoma; IMRT, intensity modulated radiotherapy; NSCLC, Non-small lung cancer; PTV, Planning Target Volume; VMAT, volumetric arc therapy.*

### Daily Position Correction

**FIGURE 2:**
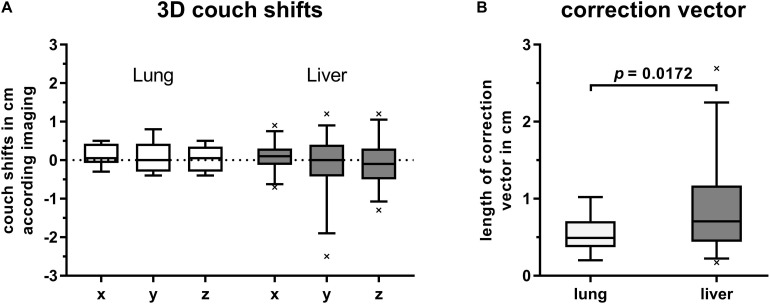
Box plots with boxes extending from 25th to 75th percentiles. Whiskers are drawn down to 5th and up to 95th percentiles, respectively. **(A)** Couch shifts are shown in left (+) to right (–), cranial (+) to caudal (–) and anterior (+) to posterior (–) directions along the *x*-, *y*-, and *z*-axis, respectively. Values were grouped by target location and derived from first CBCTs for position verification after alignment of current DIBH surface to that of the reference surface. **(B)** Length of correction vectors of first CBCTs for image-guidance in DIBH and grouped by target location. Statistical difference was calculated by Welch’s *t*-test.

Despite correct alignment of patients’ surface to the reference surface in DIBH, a subsequent CBCT-based image correction was required for most treatment fractions. These couch shifts ranged from −7 to 9 mm, −25 to 12 mm, and −13 to 12 mm in lateral, longitudinal and vertical direction, respectively. Nevertheless, median values of all shift directions of first CBCT were close to zero. Grouping patients by target location revealed higher shifts for tumors in the liver compared to lung targets. Highest ranges were observed for shifts in cranial-caudal direction along the *y*-axis (Figure 2A).

The mean lengths of applied position correction vectors were 5.4 mm (2.0–10.2) and 8.8 mm (1.7–26.9) for lung and liver targets, respectively. Compared to liver targets, the correction vectors of lung targets were significantly smaller (*p* = 0.0172, Welch’s *t*-test, Figure 2B).

A second position verification by CBCT in DIBH utilizing the newly captured reference surface could confirm a correct set-up in most cases. A new couch shift was required in 1 of 9 (11%) and 7 of 34 (21%) treatment sessions for lung and liver targets, respectively. Despite higher rates for liver SBRT, there was no significant difference in the frequency of the need for a second position correction for lung and liver targets (*p* = 1.0, Fisher’s exact test).

### Intra- and Interfractional Variability

**FIGURE 3:**
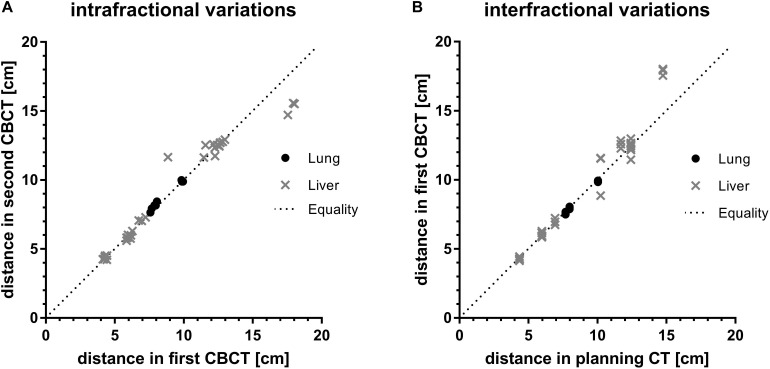
Assessment of **(A)** intra-and **(B)** interfractional variations by comparison of absolute distances between a breathing independent point to a breathing dependent movable point of first compared to second CBCT and of planning CT compared to first CBCT for every fraction, respectively. Values are grouped by target location. The dotted line indicates theoretical equality of distances in imaging.

The planning CT and the two CBCTs acquired for every treatment session and each patient were retrospectively analyzed for intra- and interfractional difference. Figure 3 shows the calculated spatial distances from an individually selected, breathing-independent point (mostly bony structures of the spine at the level of the target) to another individually chosen, breathing-dependent point near the gross tumor (mostly surgical clips or a vessel branching) for each patient and CBCT.

In case of a perfect match, the distances between the breathing-independent point and the breathing-dependent point were equal for every imaging series (planning CT and CBCT). Equality of distances of first and second CBCT as well as of planning CT and first CBCT would correspond to no intra- and no interfractional difference, respectively.

Intra-fraction distances of both lung and liver targets were close to the line of equality (Figure 3A). On average, the intrafractional differences were 1.6 mm (-3.9 to 0.5) and 1.2 mm (-27.8 to 28.3) for targets in the lungs and liver, respectively. Despite higher ranges for liver targets compared to lung targets the interquartile ranges (IQR) for both lung and liver targets were similar (2.5 mm vs. 2.8 mm). There were no significant differences of intrafractional spatial distances between lung and liver target location (*p* = 0.12, Welch’s *t*-test).

In contrast, mean interfractional differences for lung targets were 0.9 mm (0.7–2.1; IQR 1.7 mm), whereas in liver targets the average difference was 3.8 mm (−32.7 to 13.8) with a higher IQR of 6.8 mm (Figure 3B). These interfractional differences were statistically significant (*p* = 0.01, Welch’s *t*-test), indicating higher variability between treatment sessions for liver SBRT compared to lung SBRT.

### Differences in Treated Volumes

**FIGURE 4:**
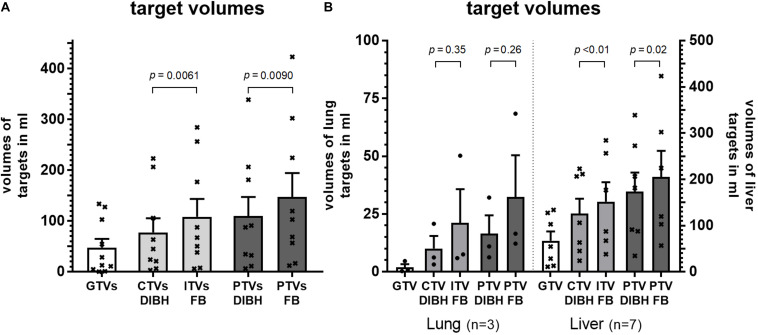
Volumes of gross tumor (GTV), clinical target volume (CTV), internal target volume (ITV) and planning target in DIBH (CTV DIBH and PTV DIBH) as well as in free-breathing (ITV FB and PTV FB) for **(A)** all patients and **(B)** grouped by target location. Statistical differences were calculated by paired *t*-test.

The mean gross tumor volumes (GTV) of lung targets, liver targets and all lesions were 1.9, 67, and 48 ml, respectively, with significantly smaller GTVs for lung targets (*p* = 0.018, Welch’s *t*-test). The mean internal target volumes (ITV) which comprise the clinical target volumes (CTV) with additional margins obtained from 4D-CT data in free-breathing was 108 ml for all lesions. By using DIBH these margins could be omitted and the CTV in DIBH was 28% smaller (77 ml, *p* < 0.01, paired *t*-test, Figure 4A). Consequently, planning target volumes of all targets could be reduced significantly by 26% from an average of 148 ml in free-breathing (FB) to 110 ml utilizing DIBH (*p* < 0.01, paired *t*-test). This reduction of target volumes by using DIBH was seen for both lung and liver targets. However, statistical significance was not reached for the smaller sub-group of lung targets (Figure 4B).

## Discussion

We share a new work-flow for SBRT in DIBH by combining SGRT and IGRT in patients with targets in the lungs and liver and demonstrate feasibility. Utilizing DIBH for SBRT significantly reduced the irradiated volumes compared to free-breathing treatment plans. SGRT enabled initial couch shifts based on first CBCT in DIBH to be close to zero, but the ranges and absolute correction vectors of liver targets were significantly higher when compared to lung targets. This interfractional variability resulted in a daily need for position correction and for an updated reference surface for most patients for every treatment session. Intrafractional movements on the other hand, were quite low with second CBCTs in DIBH rarely requiring correction of patient positioning in both liver and lung SBRT. Most liver SBRT patients exhibited reproducible intrafractional positioning in DIBH. In contrast to targets in the liver, both intra- and interfractional variability of lung targets was small indicating a better correlation of patients’ surface to lung targets rather than to liver targets.

Deep inspiration breath-hold was introduced many years ago for radiotherapy of left sided breast cancer patients to reduce the irradiated heart volume by enlarging the distance of the heart to the chest well (15). Reproducibility was ensured by the active breathing coordinator^TM^ (ABC^TM^, Elekta, Stockholm, Sweden), a commercial device consisting of a nose clamp and a mouthpiece that is connected to a breathing tube containing a valve which closes once a pre-defined target air volume is inhaled by the patient. The prospective United Kingdom HeartSpare Study compared this ABC^TM^-assisted breath-hold with a voluntary breath-hold technique and found that the latter was non-inferior in terms of reproducibility and normal tissue sparing. Moreover, voluntary breath-hold was faster and preferred by both patients and therapists (16).

Surface-guided radiotherapy describes the use of commercially available surface imaging solutions that were primarily developed to assist with patient set-up before radiotherapy delivery. These tools precisely indicate the spatial difference of a region of interest (ROI) on the patients’ body surface and a corresponding reference surface generated from the external contour of the planning simulation CT, facilitating a fast and accurate patient set-up with six degrees of freedom (17). SGRT is non-invasive, patients are not exposed to additional radiation dose, it basically does not rely on skin marks and allows monitoring of DIBH with most data available for SGRT with DIBH for adjuvant radiotherapy in left sided breast-cancer (18). Since SGRT allows not only monitoring of the patient-setup and breathing before, but also during treatment it is of particular interest for SBRT to ensure a safe, accurate and precise dose delivery during the entire treatment session.

For SBRT in targets that move with respiration, an abdominal compression is traditionally employed to mitigate breathing motion and to decrease the residual range of motion, as assessed during time resolved CT acquisition. These 4D-CT data are used to create a PTV that encompasses the tumor during the entire breathing cycle or to define a specific gating window which results in reduced irradiation volumes but, on the other hand, extend treatment delivery time (4). Several reports, however, have commented that the 4D-CT information from planning is not necessarily representative of the motion amplitude during treatment (19, 20). Moreover, breathing patterns may change both within and between treatments (intra- and interactionally) (21, 22). Therefore, we tested the feasibility of combining SGRT with IGRT to enable SBRT in DIBH.

Earlier data on SGRT and SBRT in free-breathing suggests that the pre-imaging treatment set-up for SBRT can be improved by SGRT compared to in-room laser localization of skin tattoos or skin marks (23). Moreover, SGRT reliably detects intrafractional shifts during treatment delivery when deviations extend 2 mm, as confirmed by CBCT (24). In addition, DIBH improves image quality and reduces craniocaudal registration uncertainties compared to free-breathing in lung cancer radiotherapy (25). Furthermore, using breath-hold for SBRT delivery reduces motion artifacts that is especially important for small lung tumors that are poorly visualized on imaging even with modern linear accelerators (26). Yet to perform IGRT in combination with DIBH, imaging within a breath-hold of approximately 30 s was required. We achieved this through our technique by not performing a complete gantry rotation and instead using a higher than standard gantry rotation speed without appreciable loss of image information. As previously suggested, faster CBCTs show no significant registration differences compared to standard CBCTs and a higher gantry velocity with fewer projections produces fewer reconstruction artifacts (27).

We observed highest interfractional variability in cranio-caudal direction and lowest in left-right direction, underpinning the idea that respiratory motion, which is mostly performed by the diaphragm, impacts most on target localization. Indeed, using SGRT for positioning of breast cancer patients in free-breathing showed least errors for lateral set-up compared to imaging (28). Our data further suggest a higher correlation of thoracic DIBH surface to intrathoracic targets than to abdominal targets, despite the fact that we chose to set the ROI for DIBH monitoring to the lower thorax for both thoracic and abdominal targets. Indeed, the correlation of skin to tumor is not necessarily constant, especially for liver and pancreas as previously reported (29, 30). In addition, lung volumes in DIBH may not necessarily always be the same for every breath-hold, although SGRT may confirm a match within a ROI on the patient’s body surface to the corresponding reference surface. Such variations in lung volumes during breath-hold were recently reported to have an impact on target localization (31).

Previous work on employing active breathing coordinator (ABC^TM^)-controlled breath-hold for lung and liver SBRT reported good intrafractional reproducibility of liver position in the majority of patients. However, interfractional reproducibility was worse, suggesting a need for daily image guidance (31–33). Another study, conducted by Lu et al., also found higher interfractional than intrafractional motion but observed clinically significant intrafractional motion >3 mm in 26 and 47% of patients with liver and lung cancer, respectively (34). This intrafractional differences could be explained by an intra-breath-hold residual motion of the diaphragm that was recently estimated by an ultrasound-based monitoring of the diaphragm dome during ABC^TM^-controlled breath-holds to be <2 mm and <5 mm in 59 and 95% of 385 DIBHs in 13 patients (35).

The most elegant solution for precise and accurate SBRT in DIBH is probably possible with MR-guidance since on-board magnetic resonance (MR) imaging in linear accelerators (MR-LINAC) for set-up and treatment delivery is non-invasive, exposes the patient to no additional radiation and allows for direct target localization and real-time visualization (36). In contrast, SGRT remains an indirect visualization of the patient’s surface although it gives feedback of localization in real-time, too. Nevertheless, MR-LINACs are currently rarely available and there are still some issues concerning reliability of gating and tracking procedures, the additional time needed for dose-optimization and the dose delivery time, that is yet mostly slower than in conventional linear accelerators (37). Thus, a SBRT session in DIBH at a MR-LINAC would require a patient to perform more breath-holds. In addition, a MR LINAC treatment is not an option for every patient depending on body size, claustrophobia and metal implants or implanted electronic devices.

## Conclusion

Surface-guided radiotherapy in DIBH for lung and liver tumors using a combination of SGRT and IGRT is feasible. This approach is easy to incorporate in contemporary practice and does not require any breathing tubes connected to the patient. Nevertheless, daily 3D imaging is of paramount importance given that interfractional variability is high, particularly in DIBH for liver SBRT.

## Data Availability Statement

The datasets presented in this article are not readily available because legal regulations disallow and as a requirement of local ethics committee clinical raw data must not be made available to others than those involved in the study. Requests to access the datasets should be directed to patrick.naumann@med.uni-heidelberg.de.

## Ethics Statement

The studies involving human participants were reviewed and approved by Ethikkommission Heidelberg. The patients/participants provided their written informed consent to participate in this study.

## Author Contributions

PN and VB were responsible for conceptualization, data curation and formal analysis. BR and JD supervised the project. PN drafted the manuscript. PN, VB, JF, BR, and ET-M participated in patient treatment. BF and JL critically reviewed the manuscript. All authors read and approved the final manuscript.

## Conflict of Interest

In the past 5 years JD attended advisory board meetings of MERCK KGaA (Darmstadt), for which the University hospital Heidelberg received travel grants and honoraria. His department further received funding for research projects and for educational grants to the University hospital of Heidelberg by Accuray (2016), Merck KGaA (2015-open), Siemens GmbH (2015-open), Viewray (2018-open), Vision RT (2017-open). He is CEO of the HIT GmbH and also member of the kuratorium of the Physikalisch Technische Bundesanstalt (PTB). As chair of HIRO (Heidelberg Institute of Radiation Oncology) and director of the NCT (National Center for Tumor Diseases) he is responsible for collaborations with a multitude of companies and institutions. JD confirms that to the best of his knowledge none of the above funding sources was involved in the data analysis and preparation of this work. The remaining authors declare that the research was conducted in the absence of any commercial or financial relationships that could be construed as a potential conflict of interest.
